# Supplementary material for: Improvement in Infection Prevention and Control Performance Following Operational Research in Sierra Leone: A Before (2021) and After (2023) Study
Source: Trop Med Infect Dis. 2023 Jul 23;8(7):376. doi: 10.3390/tropicalmed8070376 (PMC10383112; doi:10.3390/tropicalmed8070376)
Supplement: Supplementary file 1 [file tropicalmed-08-00376-s001.zip › Supplementary Table S1.pdf]

Supplementary Table S1: Median percentage scores of the core components of IPCAF in twelve district secondary hospitals of Sierra Leone during 20121 and 2023

District Secondary Hospitals	Year	i. IPC programme	ii. IPC guidelines	iii.IPC education & training	iv. HAI surveillance	v. Multimodal strategies	vi. Monitoring/ audit and feedback	vii. Workload, staffing, bed occupancy	viii. Built environment, materials and equipment	*Cumulative score	#Cumulative median score (%)
DSH1	2021	50	68	85	43	45	63	30	61	444	55
	2023	58	78	40	48	40	65	35	51	414	52
DSH2	2021	50	75	85	43	45	50	35	63	445	56
	2023	48	70	70	45	35	60	45	46	419	52
DSH3	2021	45	75	75	28	45	48	30	49	394	49
	2023	73	78	70	40	40	48	50	51	449	56
DSH4	2021	48	68	80	45	45	65	25	54	429	54
	2023	68	80	80	40	55	55	45	46	469	59
DSH5	2021	35	68	60	45	45	58	40	50	400	50
	2023	63	80	85	50	55	70	45	76	524	65
DSH6	2021	30	68	65	45	45	53	25	51	381	48
	2023	50	80	75	50	60	58	50	83	505	63
DSH7	2021	28	68	80	45	45	58	25	49	396	50
	2023	78	88	80	50	35	48	45	55	478	60
DSH8	2021	48	68	80	50	45	50	25	55	420	53
	2023	58	70	50	48	25	48	35	58	390	49
DSH9	2021	50	75	80	5	45	58	30	51	394	49
	2023	73	78	85	63	70	75	40	75	558	70
DSH10	2021	28	68	65	45	45	48	45	55	398	50
	2023	75	80	85	48	50	63	45	69	514	64
DSH11	2021	40	68	80	50	45	53	25	50	410	51
	2023	58	80	75	45	35	38	35	61	426	53
DSH12	2021	25	65	70	10	45	30	30	51	326	41
	2023	68	73	80	43	75	58	35	59	489	61

Abbreviation: DSH= District hospital, IPC= Infection, Prevention and Control; HAI= Healthcare Associated Infection. * Maximum score for each component is 100 and for the cumulative it is 800, # Percentages are calculated relative to the maximum score for the component. Grade: IPC performance in each component will be graded based on the obtained percentage: i) inadequate (0%-25%) ii) basic (25.1%-50%) iii) intermediate (50.1%-75%) and iv) advanced (75.1%-100%)
